# Zika virus infection and congenital anomalies in the Americas: opportunities for regional action

**DOI:** 10.26633/RPSP.2017.174

**Published:** 2017-12-12

**Authors:** Mariela Larrandaburu, Fernanda Sales Luiz Vianna, André Anjos da Silva, Luis Nacul, Maria Teresa Vieira Sanseverino, Lavínia Schuler-Faccini

**Affiliations:** 1 Postgraduate Program in Genetics and Molecular Biology Universidade Federal do Rio Grande do Sul Porto AlegreRio Grande do Sul Brazil Postgraduate Program in Genetics and Molecular Biology, Universidade Federal do Rio Grande do Sul, Porto Alegre, Rio Grande do Sul, Brazil.; 2 London School of Hygiene and Tropical Medicine School London School of Hygiene and Tropical Medicine School London United Kingdom London School of Hygiene and Tropical Medicine School, London, United Kingdom.

**Keywords:** Zika virus, microcephaly, epidemiological surveillance, Americas, Virus Zika, microcefalia, vigilancia epidemiológica, Américas, Zika virus, microcefalia, vigilância epidemiológica, Américas

## Abstract

*The Zika virus (ZIKV) was identified in 1947 in the Zika forest in Uganda, but recently it has emerged as a public health threat. The first evidence of human infection occurred in 1952, but only in April 2007 was the first outbreak in humans recognized. In the Americas, a ZIKV outbreak began in Brazil in 2015, and from the second half of 2015 onward, a substantial number of newborns with severe microcephaly began to be reported to health authorities. In February 2016, the World Health Organization (WHO) declared that the clusters of microcephaly cases in areas affected by ZIKV constituted a Public Health Emergency of International Concern. Seldom has there been such a resultingly vast production of scientific literature in record time. In this report we discuss the impact of ZIKV infection during pregnancy, the diagnosis and surveillance of microcephaly, the recognition of a clinical phenotype of ZIKV congenital infection, and opportunities for public health action. We consider this to be a unique opportunity for countries in the Region of the Americas to develop, strengthen, and improve surveillance systems for congenital anomalies and teratogen information services. Creating health needs assessment tools for low- and middle-income countries may help them to develop effective policies to ensure primary, secondary, and tertiary prevention measures for congenital anomalies. Such initiatives will be useful for ZIKV congenital syndrome control and also for having a much wider impact on a significant proportion of preventable and manageable congenital conditions*.

## RESUMEN

El virus del Zika (ZIKV) se identificó en 1947 en el bosque de Zika, en Uganda, pero recientemente ha surgido como una amenaza para la salud pública. Los primeros datos de la infección en seres humanos surgieron en 1952, pero no fue hasta abril del 2007 que se reconoció el primer brote en seres humanos. En la Región de las Américas, en el 2015 se inició un brote del virus en el Brasil, y desde la segunda mitad de ese año en adelante se empezó a notificar a las autoridades de salud un número considerable de recién nacidos con microcefalia grave. En febrero del 2016, la Organización Mundial de la Salud (OMS) declaró que los conglomerados de casos de microcefalia detectadas en las zonas afectadas por el ZIKV constituían una emergencia de salud pública de importancia internacional. Raramente ha habido una consiguiente producción tan vasta de publicaciones científicas en un tiempo récord. En este informe se aborda la repercusión de la infección por el ZIKV durante el embarazo, el diagnóstico y la vigilancia de la microcefalia, el reconocimiento de un fenotipo clínico de la infección congénita por el ZIKV y las oportunidades para las intervenciones de salud pública. Consideramos que se trata de una oportunidad única para los países de la Región de las Américas de desarrollar, fortalecer y mejorar los sistemas de vigilancia de las anomalías congénitas y los servicios de información sobre teratógenos. La creación de herramientas de evaluación de las necesidades de salud para los países de ingresos bajos y medianos puede ayudarles a elaborar políticas eficaces destinadas a asegurar medidas preventivas primarias, secundarias y terciarias para las anomalías congénitas. Dichas iniciativas serán útiles para el control del síndrome congénito del ZIKV y también para tener una incidencia mucho mayor sobre una proporción significativa de las enfermedades congénitas prevenibles y controlables.

## RESUMO

O vírus Zika (ZIKV) foi identificado em 1947 em animais na floresta de Zika, em Uganda, mas se tornou um grande risco à saúde pública nos últimos anos. A primeira evidência de infecção humana data de 1952, porém o primeiro surto em seres humanos foi registrado somente em abril de 2007. Na Região das Américas, o Brasil registrou um surto de zika em 2015 e, a partir de meados daquele ano, passou a ser notificado aos órgãos de saúde um número considerável de casos de recém-nascidos com microcefalia grave. Em fevereiro de 2016, a Organização Mundial da Saúde (OMS) decretou que a concentração de casos de microcefalia nas áreas de ocorrência de zika representava uma situação de emergência em saúde pública de interesse internacional. Raras vezes se viu tamanha produção de conhecimento científico em tão pouco tempo. Este artigo examina as consequências da infecção pelo vírus Zika durante a gestação, discorre sobre o diagnóstico e a vigilância de casos de microcefalia e a identificação de um fenótipo clínico da infecção congênita pelo ZIKVS e aponta oportunidades para ação em saúde pública. Os autores consideram ser esta uma oportunidade única aos países da Região das Américas de expandir a capacidade e reforçar e melhorar a qualidade dos sistemas de vigilância de malformações congênitas e os serviços de informação sobre teratogenicidade. Desenvolver instrumentos para avaliar as necessidades em saúde dos países de baixa e média renda pode favorecer a formulação de políticas eficazes que garantam medidas de prevenção primária, secundária e terciária de malformações congênitas. Tais iniciativas possibilitariam o controle da síndrome congênita do zika e também poderiam repercutir mais amplamente em um conjunto importante de afecções congênitas que podem ser prevenidas e controladas.

On 1 February 2016, the World Health Organization (WHO) declared that the clusters of microcephaly cases and other neurological disorders such as Guillain-Barré syndrome in some areas affected by Zika virus (ZIKV) constituted a Public Health Emergency of International Concern (PHEIC) (1). “The increased prevalence of microcephaly at birth is particularly alarming, because it is a painful burden on the families and communities,” pointed out WHO Director-General Margaret Chan (2).

The Zika virus was identified in the 1940s in the Zika forest in Uganda in monkeys. The first evidence of infection in humans occurred in 1952. Some five decades later, the international community recognized that the first ZIKV outbreak in humans had occurred in April 2007 in Yap, one of the states of the Federal States of Micronesia, in the Pacific Ocean. At that time, the transmission was reported in 10 other Pacific island countries and areas (3). In the Americas, the virus entered through Brazil, probably in 2013 (3). By November 2016, 48 countries in the Americas had reported autochthonous cases (4), with the *Aedes aegypti* mosquito being the most significant vector (5).

In this report we discuss the impact of ZIKV infection in pregnancy, the diagnosis and surveillance of microcephaly, the recognition of a clinical phenotype of ZIKV congenital infection, and opportunities for public health action in countries in the Americas.

## HOW THE MICROCEPHALY OUTBREAK WAS DETECTED

In early 2015, Brazil’s Ministry of Health began to receive reports of cases of an eruptive condition of unknown cause, especially in states in the Northeast region (6), and by April 2015, ZIKV was detected in those patients (7). The clinical manifestations were mild, with many regressing spontaneously without any clinical intervention. From the second half of 2015 onward, cases of newborns with microcephaly (8) and eye abnormalities began to be reported (9).

Microcephaly is defined as a smaller than expected occipital-frontal head circumference (OFHC) for gender and gestational age. Since the growth of the cranium depends on the forces of the expanding brain, microcephaly is a measure of brain development. Measurement of newborn OFHC is a screening tool for detecting microcephaly independently from its cause. This condition is a clinical finding that can manifest in isolation or as part of different syndromes caused by genetics (genetic or chromosomal) or environmental factors.

In Brazil, between October 2015 and November 2016, 10 276 cases of microcephaly were reported in accordance with the definition of the Health Ministry’s surveillance protocol, which includes newborns and fetal losses. Of these, 3 113 (30.3%) of the cases remain under investigation at the time of this manuscript submission, 2 189 cases (21.3%) have been confirmed for microcephaly and/or central nervous system abnormalities suggestive of congenital infection, and 4 974 cases were ruled out (10). In 2010, the Brazilian Live Birth Information System (SINASC) reported a prevalence of microcephaly in Brazilian newborns of 5.7 cases per 100 000 live births (11). However, according to the Latin American Network of Congenital Malformations (ECLAMC), in Brazil there had been no reliable data on microcephaly before the Brazilian epidemic, even for those cases considered to be severe. Thus, at this time, the focus on this anomaly could lead to overreporting and misdiagnosis (12).

## CLINICAL PHENOTYPE AND THE RECOGNITION OF A NEW TERATOGENIC SYNDROME

In Brazil, by one year after the ZIKV outbreak, more the 1 500 cases of congenital microcephaly associated with maternal ZIKV infection had been described. The phenotype can be defined as an “embryo-fetal brain disruption sequence by the Zika virus” (EFDS-ZIKV). The phenotype involves severe cerebral lesions and a dysmorphic spectrum ranging from babies with mild/moderate to severe microcephaly secondary to the brain disruption sequence, and often neurological signs of cortical damage such as hypertonia and arthrogryposis (13).

Table 1 summarizes the clinical findings (from neuroimaging and pathology) from the reported cases, most of which were autochthonous infections in Brazil. Two reports are directed towards the neuroimaging findings, and two others have relevant information about the fetal postmortem findings. It is now acknowledged that microcephaly is perhaps only part of the spectrum of congenital ZIKV infection, where some babies born without microcephaly have manifested neurological abnormalities only after the neonatal period (14).

## EPIDEMIOLOGICAL SITUATION OF ZIKA VIRUS AND SURVEILLANCE SYSTEMS FOR CONGENITAL ANOMALIES IN THE AMERICAS

By November 2016, 48 countries/territories of the Americas had already confirmed cases of autochthonous ZIKV infection (increasing from 34 at the beginning of 2016). Besides Brazil (with more than 1 500 cases), 20 countries had reported congenital syndromes associated with Zika virus: Colombia (58 cases); the United States of America (31); Guatemala (15); French Guiana and Martinique (14 each); the Dominican Republic (10); Panama (5); El Salvador and Puerto Rico (4 each); Plurinational State of Bolivia (3); Costa Rica, Honduras, Paraguay, and Suriname (2 each); and Argentina, Canada, Grenada, Guadeloupe, Haiti, and Trinidad and Tobago (1 each).

Thirteen countries/territories in the Region of the Americas (Brazil, Colombia, the Dominican Republic, El Salvador, French Guiana, Guadeloupe, Guatemala, Honduras, Jamaica, Martinique, Puerto Rico, Suriname, and Venezuela) have reported an excess of Guillain-Barré syndrome (GBS), with at least one case involving laboratorial confirmation of ZIKV (5, 14). Before 2015, only 11 countries in the Americas had a surveillance system for congenital anomalies (SSCA): 5 in the Andean Region and Southern Cone (Argentina, Brazil, Chile, Colombia, Uruguay), 3 in the Caribbean and Central America (Costa Rica, Cuba, Puerto Rico), and 3 in North America (Canada, Mexico, United States). After the ZIKV outbreak in 2015, 8 countries started an SSCA program: 3 in the Andean Region (Ecuador, Paraguay, Peru) and 5 in the Caribbean and Central America (Dominica, the Dominican Republic, El Salvador, Nicaragua, Panama).

Other modes of transmission, such as sex, blood transfusions, and organ transplants, may contribute (albeit on a much smaller scale) to the spread of the disease (15).

Table 2 summarizes the main characteristics of congenital anomaly surveillance systems, along with the baseline for microcephaly prevalence at birth in Latin America before the ZIKV epidemic in 2015. Brazil, for example, has had the nationwide Live Birth Information System (SINASC). The certificate of live birth is compulsory, and the presence of congenital anomalies must be registered on it. It was precisely this system that, despite its many weaknesses (mainly underreporting), enabled the confirmation of the microcephaly outbreak in Brazil, which had been initially notified by pediatric neurologists. Costa Rica and Uruguay have had population-based systems for the surveillance of congenital anomalies, while other countries have had hospital-based systems, one of which includes data from various South American countries.

**TABLE 1.** Clinical phenotype of Zika virus embryopathy in confirmed and suspected cases

Clinical, neuroimaging, and pathological findings	Published studies and case reports (no. of cases)										
	A (35)	B (3)	C (4)	D (1)	E (1)	F (2)	G (12)	H (4)	I (13)	J (23)	K (13)
Microcephaly	X	X	X	X	X	X	X	X	X	X	ND^a^
Corpus callosal anomaly	X	ND	X	ND	X	X	X	ND	X	X	X
Vermian anomaly	X	ND	X	ND	ND	X	ND	ND	ND	ND	ND
Variable ventricular dilatation	X	ND	X	X	X	X	X	ND	X	X	X
Cerebral calcifications	X	X	X	X	X	X	X	X	X	X	X
Brain atrophy	X	ND	ND	ND	X	X	ND	ND	ND	X	ND
Enlarged cisterna magna	X	ND	ND	ND	ND	X	ND	ND	ND	X	X
Hypoplasia of the brain stem and spinal cord	ND	ND	ND	X	ND	ND	ND	ND	ND	X	X
Neuronal migration disorders	X	ND	X	X	X	X	X	ND	X	X	ND
Variable gliosis	ND	ND	ND	ND	X	ND	ND	X	ND	ND	ND
Microphthalmia	X	ND	ND	ND	ND	ND	ND	ND	NA^b^	NA	NA
Alterations in the macula and gross macular pigment mottling	ND	X	ND	ND	ND	ND	ND	ND	NA	NA	ND
Fovea reflex loss	ND	X	ND	ND	NA	ND	ND	ND	NA	NA	ND
Hypertonia, spasticity, seizures, hyperreflexia, irritability, tremors	X	ND	ND	ND	NA	ND	ND	ND	NA	NA	X
Oligohydramnios and anhydramnios	ND	ND	ND	ND	ND	ND	X	ND	NA	NA	ND
Abnormal arterial flow in the cerebral or umbilical arteries	ND	ND	ND	ND	ND	ND	X	ND	NA	NA	ND
Intrauterine growth restriction	ND	ND	ND	ND	ND	ND	X	ND	NA	NA	ND
Elective termination of pregnancy	NA	NA	NA	X	X	NA	NA	NA	NA	NA	NA

a ND = not described.

b NA = not applicable.

***Source:*** Prepared by the authors from information in items included in this article’s References listing and in other sources: **A**: (8); **B**: (9); **C**: Jouannic JM, Friszer S, Leparc ND, Goffart I, et al. Zika virus infection in French Polynesia. Lancet. 2016 Mar 12;387(10023):1051-2.; **D**: Mlakar J, Korva M, Tul N, Popović M, Poljšak-Prijatelj M, Mraz J, et al. Zika virus associated with microcephaly. N Engl J Med. 2016 Mar 10;374(10):951-8.; **E**: Driggers RW, Ho CY, Korhonen EM, Kuivanen S, Jääskeläinen AJ, Smura T, et al. Zika virus infection with prolonged maternal viremia and fetal brain abnormalities. N Engl J Med. 2016 Jun 2;374(22):2142-51.; **F**: Oliveira Melo AS, Malinger G, Ximenes R, Szejnfeld PO, Alves Sampaio S, Bispo de Filippis AM. Zika virus intrauterine infection causes fetal brain abnormality and microcephaly: tip of the iceberg? Ultrasound Obstet Gynecol. 2016 Jan;47(1):6-7.; **G**: (13); **H**: Martines RB, Bhatnagar J, Keating MK, et al. Notes from the field: evidence of Zika virus infection in brain and placental tissues from two congenitally infected newborns and two fetal losses Brazil. MMWR Morb Mortal Wkly Rep 2016;65:159–60.; **I**: Cavalheiro S, Lopez A, Serra S, Da Cunha A, da Costa MD, Moron A, et al. Microcephaly and Zika virus: neonatal neuroradiological aspects. Childs Nerv Syst. 2016 Jun;32(6):1057-60.; **J**: de Fatima Vasco Aragao M, van der Linden V, Brainer-Lima AM, Coeli RR, Rocha MA, Sobral da Silva P, et al. Clinical features and neuroimaging (CT and MRI) findings in presumed Zika virus related congenital infection and microcephaly: retrospective case series study. BMJ. 2016 Apr 13;353:i1901.; **K**: van der Linden V, Pessoa A, Dobyns W, Barkovich AJ, Júnior HV, Filho EL. Description of 13 infants born during October 2015-January 2016 with congenital Zika virus infection without microcephaly at birth – Brazil. MMWR Morb Mortal Wkly Rep. 2016 Dec 2;65(47):1343–8.

It is important to note that in 2010 the 63rd World Health Assembly adopted a resolution urging countries to develop systems and to train staff in relation to the prevention of congenital anomalies, as well as to strengthen surveillance systems (16). The resolution also called on countries to provide appropriate health care for people with congenital anomalies and for their families. This step has been neglected in public agendas, despite the efforts of nongovernmental organizations, health professionals, and health authorities.

Beginning with the microcephaly outbreak in Brazil (6), and in accordance with WHO recommendations, several countries in the Americas began to implement actions focused on detecting ZIKV infections and microcephaly and other congenital anomalies. These actions by the ministries of health have mainly included training health professionals and developing and strengthening guidelines and registers. Another challenge has been to integrate different health functions that are generally conducted by separate departments in the health system, such as surveillance, prevention, and assistance related to Zika virus infections and the occurrence of microcephaly. The example of Brazil is illustrative: meetings and discussions led to production of a publication on integrated protocols for facing Zika virus infections, including surveillance and management of care for pregnancies and for affected infants (the latest version of this document is available at: http://portalarquivos.saude.gov.br/images/pdf/2016/dezembro/12/orientacoes-integradas-vigilancia-atencao.pdf).

## PUBLIC HEALTH IMPORTANCE AND ACTION

The ZIKV outbreak in the Americas began in Brazil and has spread throughout the Region of the Americas. In contrast to other infections caused by arboviruses transmitted by the same vector (*A. aegypti*), such as dengue, chikungunya, and yellow fever (which can be fatal), ZIKV has mild symptoms in the affected person but has a devastating teratogenic potential (17, 18). This situation is quite critical for families, as well as a challenge for health systems. The technology available has already enabled recognition of the genomic sequence of ZIKV, which may help explain the epidemiological findings and the neurotropism. The surveillance systems for communicable diseases are on maximum alert, and various government actions at the regional level have been taken in an attempt to reverse the current lack of data, improve information, and develop a common strategic plan to control the epidemic.

**TABLE 2.** Characteristics of congenital anomaly surveillance systems in Latin America, and the baseline of microcephaly prevalence before 2015

Country/Countries	Name of surveillance program	Year started	Legislation/Funding	Mandatory	Sources of ascertainment	Size and coverage	Stillbirth included	Verbatim description of congenital anomalies	Baseline microcephaly per 10 000 births (before 2015)
Argentina	National Registry of Congenital Anomalies of Argentina (RENAC)	2009	Funded by Ministry of Health of Argentina	No	The detection period lasts until discharge from the hospital	300 000 annual births	Yes	Yes	1.90^a^
Brazil	Live Birth Information System (SINASC)	2001	Financed by Ministry of Health of Brazil; obligatory notification	Yes	Live birth certificate	National	No	No	0.57^b^
Chile	Regional Register Congenital Malformation of Maule Health Service	2001	Based on ECLAMC and funded by the Maule Health Service	No	Pediatricians and midwives at the delivery units of participating hospitals	Maule Region; 13 500 births annually	Yes	Yes	1.4^c^
Colombia	Bogota Congenital Malformations Surveillance Program	2006	Based on ECLAMC; founded by District Health Secretary of Bogotá and Pontificia Universidad Javeriana	No	Control-case methods same as the ECLAMC (nurses, gynecologists, neonatologists)	104 700 births annually were monitored in the city of Bogotá	Yes	Yes	2.5^d^
Costa Rica	Costa Rican Birth Defects Register Center	1986	Founded by Public Health Ministry of Costa Rica; obligatory notification	Yes	Since 2009, the age of obligatory notification was extended to children under 1 year of age	The program is population based	Yes	Yes	4.31^e^
Cuba	Cuban Register of Congenital Malformation	1985	Financed by Public Health Ministry of Cuba	No	Reports are obtained from hospitals all over Cuba	96% of Cuban births	Yes	Yes	0.22^f^
Mexico	Mexican Registry and Epidemiological Surveillance of External Congenital Malformations	1978	Research grants	No	Reports are obtained from 21 hospitals in 11 cities of Mexico	3.5% of all births in Mexico	Yes	Yes	2.01^f^
Uruguay	National Registry of Congenital Defects and Rare Diseases (RNDCER)	2011	Financed by Public Health Ministry of Uruguay; obligatory notification	Yes	Reports are obtained from different health professional and patient organizations	The program is population based	Yes	Yes	0.52^g^
Various South American countries	Latin American Collaborative Study of Congenital Malformations (ECLAMC)	1968	Research program	No	Collaborating pediatricians at the delivery units of participating hospitals	Approximately 1% of the annual births of the South American countries	Yes	Yes	4.83^f^

a Available from: http://www.msal.gob.ar/images/stories/bes/graficos/0000000581cnt-reporte_operativo_renac_2014.pdf Accessed 12 May 2016.

b Available from SINASC, at: http://www2.datasus.gov.br/DATASUS/index.php?area=060702 Accessed 27 June 2016.

c Personal communication (Drs. Aurora Canessa and Rosa Fajardo) (Maule Register – ECLAMC).

d Available from: http://www.anomaliascongenitas.org/app/webroot/blog/wp-content/uploads/2015/03/Word-Anual-SEMIFINAL-.pdf Accessed 12 May 2016.

e Available from: https://www.ministeriodesalud.go.cr/index.php/vigilancia-de-la-salud/normas-protocolos-y-guias/2995-protocolo-de-vigilancia-microcefalia-en-rn-el-marco-de-la-vigilancia-del-virus-del-zika-en-costa-rica-version-final-docx/file Accessed 12 May 2016.

f Available from: http://www.icbdsr.org/filebank/documents/ar2005/Report2013.pdf Accessed 12 May 2016.

g Available from: http://www.msp.gub.uy/marco-normativo/registro-nacional-de-defectos-cong%C3%A9nitos-y-enfermedades-raras Accessed 12 May 2016. (unpublished information: total births, 191 820 (2011-2014), with 10 cases reported).

***Source:*** Prepared by the authors from the study results.

With ZIKV, there is no safe period in pregnancy, and babies can be adversely affected in all pregnancy trimesters (13). Therefore, the WHO has issued recommendations about monitoring infection by ZIKV and its consequences, including actions for surveillance and notification of cases of microcephaly and GBS. These recommendations differ according to a country’s situation: a) with epidemic transmission of ZIKV; b) with possible endemic transmission of the virus; c) at risk of transmission of the virus; and d) with no risk or low risk of transmission of ZIKV by vector mosquitoes.

In general, in line with recommendation that the WHO has made to member states, some of the indicators that should be reported are: 1) number of ZIKV cases; 2) number of ZIKV deaths; 3) number of GBS cases and deaths; 4) number of microcephaly cases; 5) number of other anomalies; 6) number of countries with autochthonous transmission; and 7) number of newly affected countries.

In addition, cases of microcephaly should continue to be monitored, accuracy of OFHC measurement in newborns should be improved, and appropriate internationally established growth curves (such as those of the INTERGROWTH-21st Consortium (19)) should be used. Finally, in countries where the epidemic is already present, it is important to have a more sensitive but less specific tool to detect brain abnormalities, such as defining microcephaly to include head circumference less than 2 *z*-scores below the mean.

Nevertheless, this more inclusive definition of microcephaly cannot be generalized. In Brazil, for example, with 3 000 000 births per year, a less strict definition of microcephaly (head circumference of 33 cm or less) led to a significant number of healthy babies being included in routine exam protocols. The health system almost collapsed due to thousands of babies being referred for clinical and laboratory protocols. Therefore, both the risks and the benefits of different criteria for inclusion of suspected cases of brain anomalies should be assessed separately for each country and each scenario. A good discussion of different diagnostic criteria for suspected microcephaly is given by Victora et al. (19).

In the countries where Zika virus infection is spreading, other congenital infections, such as syphilis, cytomegalovirus, and toxoplasmosis, are also prevalent, but rarely diagnosed early. Microcephaly can also be secondary to prenatal alcohol consumption, which is also not diagnosed in the neonatal period. Therefore, surveillance systems that are sensitive to microcephaly will have the benefit of also diagnosing other preventable cases of this congenital defect. The focus should be on prevention, early detection, and management of cases and evaluation of health needs to help prioritize actions. Currently, there are cohorts of infants and pregnant women who are being followed very closely by the health systems of different countries in the Americas. This active search for congenital anomalies will uncover many more cases of microcephaly and other abnormalities that would otherwise be missed. Upon being detected by the health system, the babies and their families should be able to count on specific early diagnosis, in line with the Convention on the Rights of Persons with Disabilities (20).

The Latin American Network of Congenital Malformations (ECLAMC) is taking the first steps to form a regional surveillance network that aims to: a) disseminate, via a Web page, the base frequencies of congenital anomalies at birth and b) provide statistical programs that any person or ministry of health can use to analyze temporal (epidemic) or geographical (endemic) frequencies (12). In response to the WHO resolution on birth defects (16), another initiative involves specific courses directed to Latin American countries on surveillance of birth defects and prematurity (see, for example: http://www.paho.org/clap/index.php?option=com_content&view=article&id=421:comprometidos-en-la-prevencion-y-vigilancia-de-anomalias-congenitas-y-partos-prematuros&Itemid=354&lang=en **).**

It is important to ensure that all these activities help develop local efforts that work toward recognizing congenital anomalies and their causes, as well as training experts in disabilities and rehabilitation. One instrument that might help low- and middle-income countries in these endeavors is the Health Needs Assessment Toolkit for Congenital Disorders, which was developed by the PHG Foundation and is freely available (http://www.bornhealthy.org/toolkit.html).

## CONCLUSIONS

Seldom has there been in the scientific literature such vast production in record time as has occurred with the congenital ZIKV infections and the microcephaly outbreak in Brazil. From the detection of cases of embryopathy by ZIKV, it is possible to delve into the etiology of microcephaly and other congenital anomalies. The neurological findings described initially may be the tip of the iceberg. We consider this to be a unique opportunity to develop, strengthen, and improve the surveillance systems for congenital anomalies, as well as teratogen information services, in the Region of the Americas. This will help develop knowledge on potential new and emerging teratogenic agents, and help establish networks to monitor indicators of progress in each country.

WHO has started coordinating efforts to define the congenital Zika virus syndrome and has issued an open invitation to all partners to join in this effort (21). Creating health needs assessment tools for low- and middle-income countries may help to develop effective policies to ensure primary, secondary, and tertiary prevention measures for congenital anomalies. In addition, until a ZIKV vaccine is available, an informed society is the best ally in effectively acting on vector control and protecting individuals. Also critical is an intersectoral policy related to housing, sanitation systems (especially for quality water supply and garbage collection), and health care. These kinds of initiatives will be useful for ZIKV congenital syndrome control, and they can also have a much wider impact on a significant proportion of preventable and manageable congenital conditions.

## Acknowledgments

The authors are grateful for technical help from Dr. Diana Valencia, of the Pregnancy and Birth Defects Team, Zika Virus Response Team, U.S. Centers for Disease Control and Prevention.

## Funding

Dr. Larrandaburu holds a Ph.D. scholarship from CAPES (Brazilian Ministry of Education).

## Disclaimer.

Authors hold sole responsibility for the views expressed in the manuscript, which may not necessarily reflect the opinion or policy of the *RPSP/PAJPH* or PAHO.
